# Iridoschisis associated with cataract: a systematic review of case reports

**DOI:** 10.31744/einstein_journal/2026RW1685

**Published:** 2025-12-01

**Authors:** Dillan Cunha Amaral, Márcio Penha Morterá Rodrigues, Guilherme Nunes Marques, Lucas Macedo Nascimento, Rafael Alonso Dinato, Vinícius Gomes Alves de Oliveira, Fernanda Galante Dourado, Guilherme da Silva Ferreira Costa, Mário Luiz Ribeiro Monteiro, Ricardo Noguera Louzada

**Affiliations:** 1 Department of Otorhinolaryngology and Ophthalmology Faculdade de Medicina Universidade Federal do Rio de Janeiro Rio de Janeiro RJ Brazil Department of Otorhinolaryngology and Ophthalmology, Faculdade de Medicina, Universidade Federal do Rio de Janeiro, Rio de Janeiro, RJ, Brazil.; 2 Faculdade de Medicina Universidade de Brasília Brasília DF Brazil Faculdade de Medicina, Universidade de Brasília, Brasília, DF, Brazil.; 3 Divisão de Oftalmologia e Laboratório de Investigação em Oftalmologia Faculdade de Medicina Universidade de São Paulo São Paulo SP Brazil Divisão de Oftalmologia e Laboratório de Investigação em Oftalmologia (LIM-33), Faculdade de Medicina, Universidade de São Paulo, São Paulo, SP, Brazil.; 4 Postgraduate Program in Surgical Sciences Faculdade de Medicina Universidade Federal do Rio de Janeiro Rio de Janeiro RJ Brazil Postgraduate Program in Surgical Sciences, Faculdade de Medicina, Universidade Federal do Rio de Janeiro, Rio de Janeiro, RJ, Brazil.

**Keywords:** Cataract extraction, Glaucoma, Iris diseases, Multimodal imaging, Cataract, Phacoemulsification

## Abstract

**Background:**

Iridoschisis is a rare, bilateral condition of unknown etiology, often associated with trauma or surgery, in which the iris stroma splits into layers. It is usually associated with glaucoma and cataract. Facectomy in these patients is challenging owing to the risk of aspirating the iris fibers and limited pupil dilation.

**Objective:**

This systematic review aimed to provide a comprehensive analysis of the clinical features, therapeutic interventions, and outcomes in patients with iridoschisis, to clarify optimal practices and identify areas for future research.

**Methods:**

We described a case of unilateral iridoschisis in a 76-year-old female, diagnosed on imaging examinations without prior ophthalmologic intervention. Preoperative planning for facectomy addressed potential surgical complications. Correct diagnosis facilitated the investigation of glaucomatous optic neuropathy, a common and sight-threatening complication if left untreated. We conducted a systematic search using Medical Subject Headings and Health Science Descriptors combined with Boolean operators.

**Sources:**

PubMed, Cochrane Library, Web of Science, and Embase were searched with no publication restrictions.

**Risk of bias:**

The risk of bias was assessed using the Critical Appraisal Checklist for case reports and case series proposed by The Joanna Briggs Institute.

**Synthesis:**

The reference lists of the retrieved studies were manually checked. A simple descriptive analysis was performed to summarize the results.

**Results:**

The search retrieved data from 234 studies. In the final analysis, 17 references were included, comprising 15 case reports and two case series. Preoperative and postoperative best-corrected visual acuities improved significantly, with cataract surgery being the most common treatment. Common complications included anterior chamber inflammation, corneal edema, and Descemet folds.

**Discussion:**

This case and systematic review provide valuable insights into the management of iridoschisis and its comorbidities, underscoring the importance of careful preoperative planning and ongoing research to refine treatment strategies.

Prospero database registration: CRD42024549865.

## INTRODUCTION

Iridoschisis is a rare manifestation of unknown etiology in which the iris stroma is cleaved into two or more layers.^( 1 )^ The anterior portion splits into strands that float in the anterior chamber.^( 1 )^ Most studies have reported a higher prevalence in women, which is usually bilateral.^( 2 )^

Although Schmitt reported a case of iridoschisis in 1922, the term was first proposed in 1945 by Loewenstein et al.^( 2 )^ Iridoschisis has no defined etiology, and studies have reported that it may represent an age-related idiopathic atrophy of the iris, a consequence of ocular trauma, prolonged use of myotic agents in glaucoma treatment, or sclerosis of the iris vessels.^( 3 )^ The age of onset of iridoschisis is usually between 60 and 70 years, which corroborates the initial hypothesis.^( 3 )^ Iridoschisis mostly occurs in the lower quadrants; however, it may diffuse in the iris.^( 4 )^ The posterior layer usually remains intact, with no changes in the sphincter or pupil dilator muscles.^( 5 )^ Iridoschisis is associated with glaucoma (primarily angle-closure), cataracts, and corneal changes.^( 4 )^ These, if present, involve the iris touching the corneal endothelium and are usually above the area of iridoschisis.^( 3 )^

The mechanism by which iridoschisis causes angle closure is unclear; nevertheless, studies have hypothesized that the strands bend forward in the anterior chamber, leading to an angle obstruction or a pupillary block due to the posterior iris pigment epithelium in the anterior capsule of the lens.^( 3 )^

Differential diagnoses include Axenfeld-Rieger and iridocorneal endothelial (ICE) syndromes.^( 4 )^ Clinical differences, such as the age of onset of clinical manifestations, appearance of the pupils, and laterality of symptoms, help exclude diagnostic hypotheses. Diagnostic imaging, such as anterior segment optical coherence tomography, may be performed, which facilitates the diagnosis and evaluation of the iridocorneal angle.^( 6 )^

The current practice for managing iridoschisis involves a combination of clinical and surgical interventions and emphasizes the prevention and treatment of glaucoma, which is frequently associated with the condition.^( 7 )^ Angle-closure glaucoma is the most common form of glaucoma in patients with iridoschisis, and other associated conditions include cataracts, lens subluxation, and corneal abnormalities.^( 8 )^ Cataract surgery in patients with iridoschisis presents unique challenges, including aspiration of iris fibrils by the phacoemulsification probe, complications during pupil dilation, and the risk of photic phenomena owing to exposure of the iris pigment epithelium.^( 9 )^ Facectomy is usually challenging.^( 10 )^ Various surgical aspects justify this, and the likely complications include aspiration of iris fibers by the phacoemulsifier or irrigation-aspiration handpiece, limited pupil dilation (possibly owing to atrophy of the pupillary margin), and injury to the sphincter muscle of the pupil.^( 5 )^ Therefore, pupillary devices are recommended, and experienced surgeons should perform surgery.^( 6 )^ Despite the progress in surgical and therapeutic techniques, the clinical characteristics, pathophysiology, and optimal management of iridoschisis remain poorly understood.

## OBJECTIVE

This systematic review aimed to provide a comprehensive analysis of the clinical features, therapeutic interventions, and outcomes in patients with iridoschisis, to clarify optimal practices and identify areas for future research.

## CASE REPORT

A 76-year-old woman presented to our outpatient clinic with a 1-year history of gradual loss of vision in the left eye (OS). Approximately 2 months prior, she underwent facectomy of the right eye (OD). The patient denied trauma, previous eye surgery for OS, or a family history of glaucoma.

**Figure 1:**
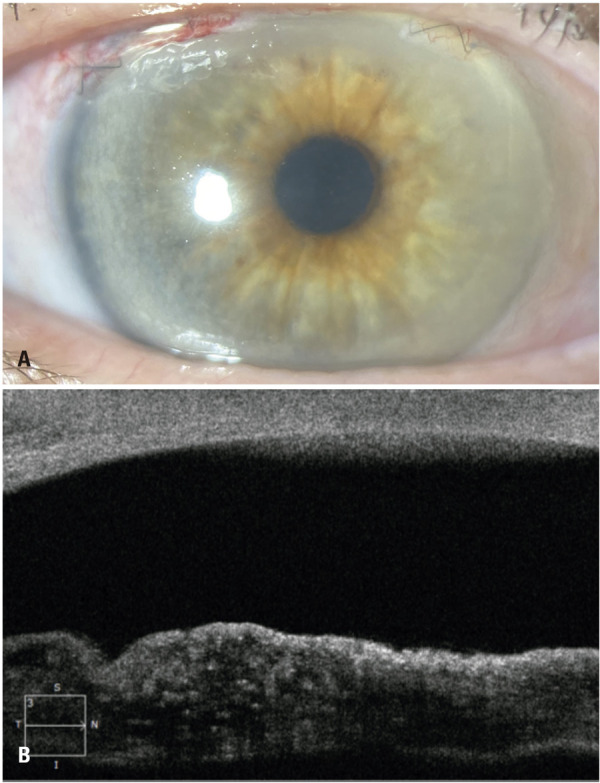
Right eye. (A) Biomicroscopy and (B) anterior segment optical coherence tomography

**Figure 2:**
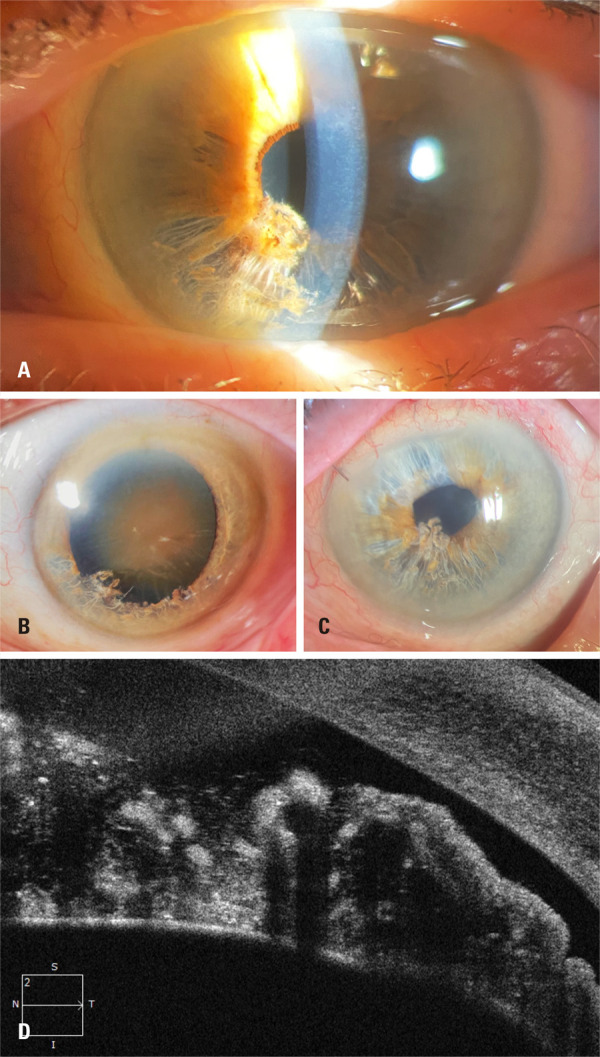
Left eye. (A) The slit-lamp indirect illumination reveals iridoschisis. Appearance of the iris: infratemporal iridoschisis and a mature cataract. (B) Biomicroscopy with a dilated pupil shows a cataract. (C) Biomicroscopy after cataract surgery, postoperative. (D) Anterior segment optical coherence tomography: Disorganization of the iris stroma corresponding to iridoschisis

The preoperative best-corrected visual acuities (BCVA) were 20/30 OD ( Figure 1 ) and hand motion OS ( Figure 2 ). Slit-lamp biomicroscopy of the OS showed a narrow-angle splitting of the anterior layers of the iris with fibrillar degeneration extending for approximately one quadrant inferiorly ( Figure 1A and C ). In addition, mature cataracts were observed ( Figure 2A and C ). Applanation tonometry indicated intraocular pressure (IOP) of 12 and 10mmHg in each eye. On gonioscopic examination, the OD showed an open angle, whereas the OS showed a narrow angle. After indentation, a pigmented trabecular meshwork was observed in all the quadrants without imprints or goniosynechia. Fundoscopic examination of the right eye revealed increased optic nerve cupping; however, examination of the left eye was impractical due to a mature cataract.

**Figure 3:**
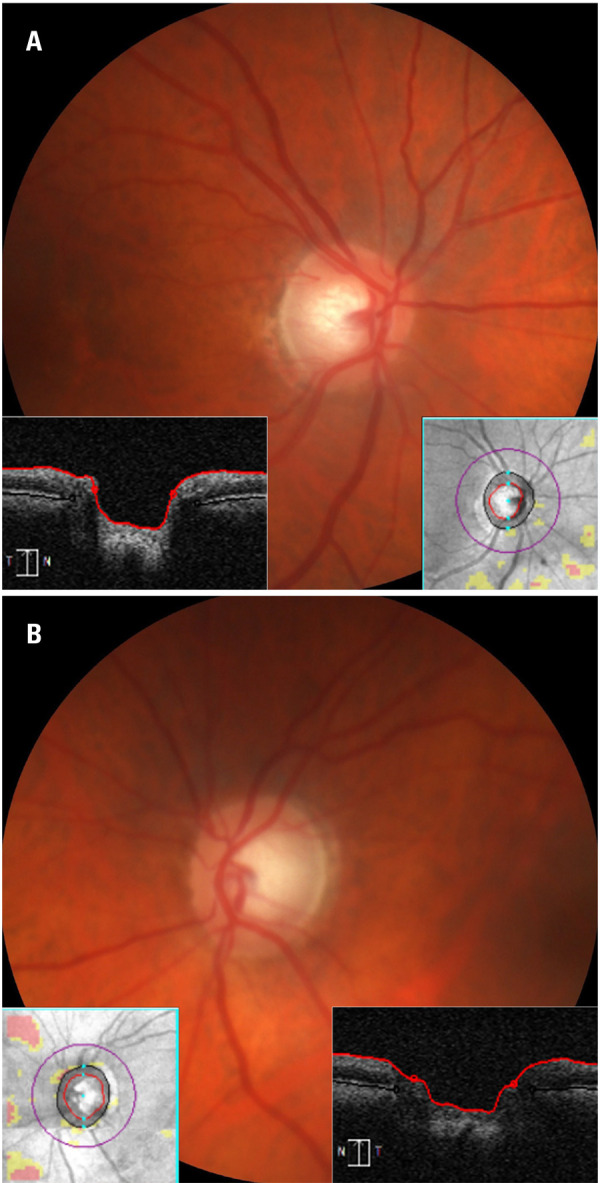
Fundus photographs and the corresponding optical coherence tomography optic nerve head scans (vertical cut) of the (A) right and (B) left eyes

The anterior segment spectral-domain OCT of both eyes showed no alterations in the OD. In the OS, cross-sectional OCT showed the presence of atrophy of the deep layers of the iris stroma, compatible with the diagnosis of iridoschisis ( Figure 2 ). Subsequently, preoperative evaluation for cataract surgery and extracapsular cataract extraction were performed under peribulbar anesthesia without pupillary devices. These eyes tended to be smaller, which could result in an elevated IOP and increased posterior pressure during surgery. Medications, such as intravenous acetazolamide or mannitol, should be readily available during the procedure to effectively manage these conditions.^( 6 )^ No postoperative complications were observed. After the postoperative follow-up, a workup for glaucoma was initiated, as the patient had asymmetry in the cupping of the optic nerve ( Figure 3 ). Retinography and OCT of the optic disc, ganglion cells, and nerve fiber layers were performed, with the latter showing no alterations.

## METHODS

### Protocol and search strategy

A systematic review was conducted according to the Preferred Reporting Items for Systematic Reviews and Meta-analysis 2020 guidelines.^( 11 )^ Four electronic databases (PubMed, Embase, Web of Science, and Cochrane) were screened from their inception until June 2024. The search strategy comprised the following terms and Boolean operators: (“iridoschisis”) AND (“phacoemulsification” OR “phaco” OR “cataract” OR “facectomy” OR “MSICS” OR “cataracts”). After the initial search, four authors independently reviewed the titles and abstracts according to the inclusion and exclusion criteria. Relevant studies were selected, and the full text was searched. Discrepancies were discussed among the authors, and a consensus was reached. The reference lists of the articles were manually searched to ensure that all potential studies were included.

### Eligibility criteria

The inclusion criteria for this study were as follows: (1) participants: individuals with iridoschisis associated with cataracts; (2) intervention: cataract surgical therapy; and (3) type of study: retrospective observational studies, case series, and case reports. The exclusion criterion was based on study type: experimental studies, reviews, abstracts, editorials, and letters to the editor were excluded.

### Outcomes and data extraction

The outcomes of interest were complications after treatment, as well as IOP, BCVA, optic discs, gonioscopy, and endothelial cell count before and after treatment. Two independent reviewers performed the data analysis and extraction, with disagreements settled by another author.

### Data extraction

The elements of data extraction included the study’s information (first author and year of publication), type of study, patients’ sex and age, follow-up, type of glaucoma, the characteristics of iridoschisis (unilateral or bilateral and location), other ocular conditions, type and details of treatment (conservative and/or surgical, mechanical dilation, and/or eye drops), and characteristics of cataracts (phakic or pseudophakic, bilateral, or unilateral). The last follow-up was used to calculate the mean difference in outcome data.

### Evaluating the risk of bias

The risk of bias was assessed using the Critical Appraisal Checklist for case reports and case series proposed by The Joanna Briggs Institute.^( 12 )^ We incorporated this information to evaluate the risk of bias in the included studies. If a case report met five of the eight appraisal criteria, it was considered acceptable and included in the systematic review. The assessments were performed by two independent reviewers.

## RESULTS

**Figure 4:**
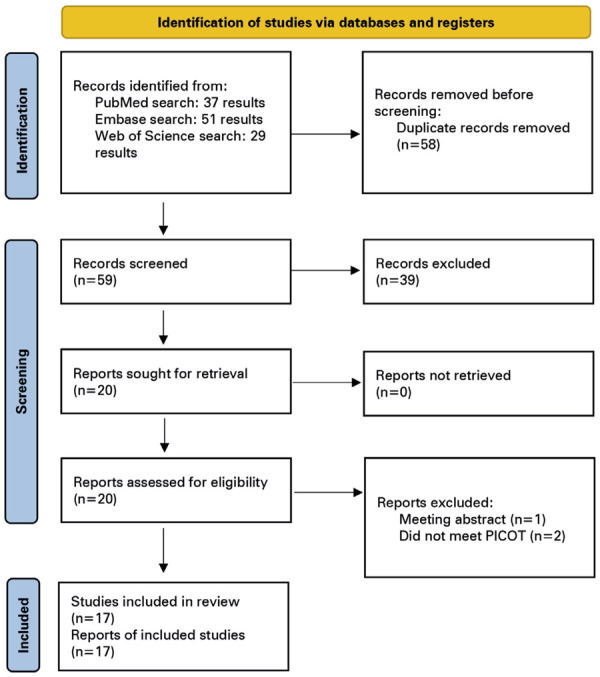

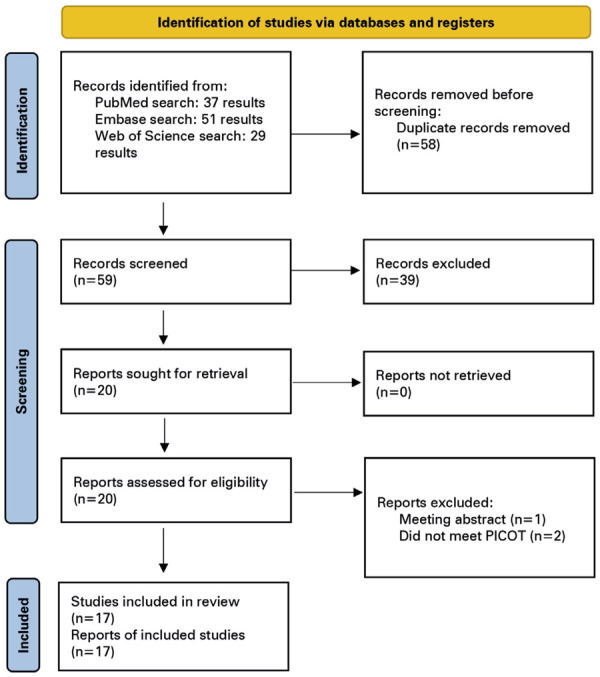
PRISMA flow diagram describing the study screening and selection process

We found 117 articles, comprising 37, 51, and 29 in PubMed, Embase, and the Web of Science, respectively. Fifty-eight non-duplicate citations were screened, and after a thorough review, 20 articles were selected after reading the abstracts for a full-text review. Three articles were excluded after full-text screening and data extraction. Finally, our systematic review revealed an additional 20 patients from 17 studies ( Figure 4 ), resulting in the inclusion of 21 patients and 35 eyes (including those of our patient) in the analysis.^( 4 , 5 , 9 , 13 - 26 )^ Among the patients, 12 were females, with an average age (mean±standard deviation [SD]) of 66.95±17.39 years. The mean follow-up period (mean±SD) for 16 of 21 case reports that explicitly stated this information was 4.18±4.66 months. The clinical findings and basic characteristics of each study are presented in table 1 , and the clinical outcomes are presented in table 2 .

**Table 1:** Baseline characteristics in the included studies

Study (Year)	Journal	Type of study	Sex	Age	Follow-up	Type of glaucoma	History of ocular trauma or hereditary ocular disease	Iridoschisis (unilateral or bilateral)	Localization of iridoschisis	Other conditions	Complications after treatment	Associated cataract: Unilateral or bilateral	Phakic or pseudophakic	Glaucoma with symmetry or asymmetry of the optic nerve compared with the iridoschisis condition	Mechanical dilation or eye drops
Aaberg et al. (2017)^(14)^	JCRS (Journal of Cataract & Refractive Surgery) Online Case Reports	Case report	Female	11 years	6 months	No glaucoma in both cases	The medical history was pertinent for severe facial and periocular eczema owing to atopic dermatitis.	Unilateral	The location of the iridoschisis is from 3 o’clock to 10 o’clock in the right eye.	External examination showed diffuse eczematous dermatologic changes on her face, including erythema, scaling, and oozing. In addition, the right eye had a milky white cataractous lens that obscured a view of the posterior segment.	There were equivocal iris changes in the left eye.	The right eye had a milky white cataractous lens that obscured a view of the posterior segment.	Phakic	N/A	N/A
Auffarth et al. (2005)^(13)^	Journal of Cataract & Refractive Surgery	Case report	Case 1: Male Case 2: Male	Case 1: 83 years/ Case 2: 73 years	N/A	No glaucoma in both cases	Case 1: Additional ophthalmic findings were nuclear cataract and pseudoexfoliation syndrome Case 2: The patient had had surgery in the left eye. The right eye presented with a brunescent cataract.	Case 1: bilateral Case 2: unilateral (right eye)	Case 1: N/A Case 2: N/A	Case 1: nuclear cataract and pseudoexfoliation syndrome Case 2: brunescent cataract	Case 1: No complications mentioned Case 2: Moderate inflammatory response in the anterior chamber, presence of Descemet folds	Case 1: Nuclear cataract, bilateral Case 2: Brunescent cataract in the right eye.	Phakic in both cases	N/A	Mechanical dilation using the Perfect Pupil device in both cases
Chen et al. (2017)^(4)^	BMC Ophthalmology	Case report	Case 1: Male Case 2: Female	Case 1: 69 years Case 2: 87 years	Case 1: 2 months Case 2: 1 month	Case 1: No glaucoma Case 2: No glaucoma	Case 1: No report Case 2: No report	Case 1: bilateral Case 2: unilateral	Case 1: Right eye - Anterior layer of the iris stroma, running in all directions Left eye: Inferior-nasal from the 5 to 9 o’clock positions Case 2: Left eye - Inferior-nasal quadrant from the 4 to 6 o’clock positions in the left eye	Case 1: Mature cataract in the right eye Case 2: Mature cataract in the right eye, iris atrophy in the inferior temporal quadrants of the left eye	Case 1: Moderate ocular hyperaemia and corneal oedema post-surgery in the right eye, improving over time Case 2: Moderate corneal edema, fibrillary materials observed in the right eye postoperatively	Case 1: Unilateral (right eye) Case 2: Unilateral (cataract was present in the right eye and had been previously treated in the left eye.)	Case 1: phakic Case 2: pseudophakic	N/A	N/A
Chen et al. (2023)^(15)^	International Journal of Ophthalmology	Brief report	Male	80 years	6 months	No glaucoma in both cases	6-month history of visual loss from both eyes	Unilateral (right eye)	The iridoschisis was observed in the right eye, where localized splitting of the iris stroma was noted, and the free ends of the atrophic iris fibers were detected in the anterior chamber.	bilateral narrow angles and cataract	No complications were mentioned	Bilateral	Phakic	N/A	Pupil-dilating eye drops were used before surgery.
**Study (Year)**	**Journal**	**Type of study**	**Sex**	**Age**	**Follow-up**	**Type of glaucoma**	**History of ocular trauma or hereditary ocular disease**	**Iridoschisis (unilateral or bilateral)**	**Localization of iridoschisis**	**Other conditions**	**Complications after treatment**	**Associated cataract: Unilateral or bilateral**	**Phakic or pseudophakic**	**Glaucoma with symmetry or asymmetry of the optic nerve compared with the iridoschisis condition**	**Mechanical dilation or eye drops**
de Paula et al. (2011)^(5)^	Revista Brasileira de Oftalmologia	Case report	Female	63 years	1 month	No glaucoma in both cases	Progressive visual acuity loss (VA) in the left eye (LE) for 1 year.	Bilateral	Both eyes (localization not specified)	Cortical cataract in the right eye, white cataract in the left eye	No complications were mentioned	Bilateral	Phakic	N/A	Pupil dilation with eye drops containing tropicamide 1%, phenylephrine 10%, and diclofenac 0.1%
Ghanem et al. (2003)^(16)^	Journal of Cataract & Refractive Surgery	Case report	Female	79 years	18 months	No glaucoma in both cases	No report	Bilateral (more intense in the right eye)	Both eyes had extensive splitting of the anterior layers of the iris with fibrillar degeneration extending from 3 to 9 o’clock, being more intense at 6 o’clock and confirming a classic iridoschisis case.	Intense corneal edema with bullous keratopathy in the right eye, approximately 3+/4+ nuclear sclerosis in the right eye, 2+/4+ nuclear sclerosis in the left eye, and diffuse atrophy of the retinal pigment epithelium in the left eye	No complications were mentioned.	Bilateral (approximately 3+/4+ nuclear sclerosis in the right eye, 2+/4+ nuclear sclerosis in the left eye)	Phakic	N/A	Pupil dilation using four drops of tropicamide 0.5% and phenylephrine 10% at 15-min intervals 1 h before surgery
Katipoğlu et al. (2023)^(17)^	International Ophthalmology	Case report	Female	79 years	There were first-week and first-month follow-ups.	No glaucoma in both cases	None reported	Bilateral	Right eye: Quadrants 2–3, 4–7, and 10–11 Left eye: Quadrants 5–7	Grade 3 senile nuclear cataract in the right eye and a grade 2 senile nuclear cataract in the left eye	No complications were mentioned	Bilateral senile nuclear cataract (Grade 3 in the right eye, Grade 2 in the left eye)	Phakic	N/A	Pupil dilatation with 2% sodium hyaluronate for anterior chamber depth and pupil dilatation; no pupil dilator was necessary
Lee et al. (2008)^(18)^	Korean Journal of Ophthalmology	Case report	Female	64 years	One week after surgery, two weeks postoperative day, and one month after surgery	No glaucoma in both cases	Approximately 6 months prior, she had undergone intracapsular cataract extraction, anterior vitrectomy, and scleral fixation of the intraocular lens in the left eye (OS).	Bilateral	Right eye (OD): Quadrants 3 to 6 Left eye (OS): Quadrants 4 to 6, diffuse atrophy from 11 to 1 o’clock	Nuclear and cortical cataract associated with posterior subcapsular cataract in the right eye (OD), pseudophakia in the left eye (OS)	Moderate inflammatory response in the anterior chamber (AC) postoperatively, Descemet folds in the cornea, traces of cells in the AC one week after surgery	Nuclear and cortical cataract associated with posterior subcapsular cataract in the right eye (OD), pseudophakia in the left eye (OS)	Pseudophakic in the left eye (OS)	N/A	Pupil dilation was performed using three drops of tropicamide 0.5% and phenylephrine hydrochloride 0.5% at 5-min intervals 1 h before surgery.
Niu et al. (2024)^(19)^	BMC Ophthalmology	Case report	Male	48 years	2 months	No glaucoma in both cases	None reported	Bilateral	Right eye: Subnasal and temporal regions Left eye: Lower part	Cataracts (both eyes), lens clouding (right eye), posterior scleral staphyloma (both eyes)	No complications were mentioned	Cataract in both eyes	Phakic	N/A	Pupil dilation using tropicamide and phenylephrine hydrochloride
**Study (Year)**	**Journal**	**Type of study**	**Sex**	**Age**	**Follow-up**	**Type of glaucoma**	**History of ocular trauma or hereditary ocular disease**	**Iridoschisis (unilateral or bilateral)**	**Localization of iridoschisis**	**Other conditions**	**Complications after treatment**	**Associated cataract: Unilateral or bilateral**	**Phakic or pseudophakic**	**Glaucoma with symmetry or asymmetry of the optic nerve compared with the iridoschisis condition**	**Mechanical dilation or eye drops**
Omoto et al. (2021)^(20)^	Case Reports in Ophthalmology	Case report	Female	76 years	3 months	No glaucoma	None reported	Bilateral	The inferior regions of both eyes	Corneal stromal edema and Descemet’s fold (both conditions were more severe in the right eye), LI holes (both eyes), moderate nuclear cataract (both eyes)	Corneal edema progression in the left eye	Cataracts in both eyes (previously managed with laser iridotomy)	Phakic	N/A	N/A
Pieklarz et al. (2020)^(21)^	American Journal of Case Reports	Case report	Male	47 years	2 weeks	Secondary glaucoma	No history of ocular trauma. The patient presented with physical features characteristic of Marfan syndrome.	Bilateral	Superotemporal iridoschisis in both eyes	Bilateral mature cataract, bilateral temporal lens subluxation	Subtle fibrin deposits on the lens surface and suspicion of UGH syndrome in the left eye.	Bilateral mature cataract	Phakic	N/A	The pupil appeared round with normal light reactions, but no specific method of dilation was mentioned.
Porteous et al. (2014)^(22)^	Clinical & Experimental Ophthalmology	Case report	Case 1: Male Case 2: Male	Case 1: 49 years Case 2: 62 years	N/A	Case 1: “secondary closure of the anterior chamber angles” Case 2: ‘’acute angle-closure’’	No reports	Case 1: Unilateral (right eye) Case 2: Unilateral (left eye)	Case 1: Significant iridoschisis in the superior and inferior iris of the right eye Case 2: inferior iridoschisis changes and crowding of the inferior angle from iris strands in the left eye	Case 1: Nuclear cataract Case 2: Cataract, non-glaucomatous optic nerve	Case 1: No complications Case 2: High postoperative cylindrical refractive error	Case 1: Unilateral (Nuclear cataract, OD - right eye) Case 2: Unilateral (OS - left eye)	Phakic in both cases	N/A	N/A
Rozenberg et al. (2004)^(23)^	Journal of Cataract & Refractive Surgery	Case report	Female	72 years	4 weeks	No glaucoma	None reported	Bilateral	Right eye: 40% of the lower anterior surface of the iris; Left eye: 25% affected area in the iris, around 6 o’clock.	Grade 3+ nuclear sclerotic cataracts were present in both eyes.	No complications	Bilateral (Grade 3+ nuclear sclerotic cataracts were present in both eyes)	Phakic	N/A	Pupillary dilation was achieved with tropicamide 1% and phenylephrine 10%, with one drop each given three times preoperatively.
Ruff et al. (2020)^(24)^	GMS Ophthalmology Cases	Case report	Male	67 years	1 month	Chronic angle closure glaucoma on the right eye	His past medical history was significant for skin basal cell carcinoma.	Bilateral	Not specified: ‘”Loose iris strands were noted on both eyes, predominantly the left eye.”	Skin basal cell carcinoma, bilateral cataracts. Advanced nuclear sclerosis and posterior subcapsular cataract were noted on the left eye.	No complications	Bilateral	Phakic	N/A	N/A
**Study (Year)**	**Journal**	**Type of study**	**Sex**	**Age**	**Follow-up**	**Type of glaucoma**	**History of ocular trauma or hereditary ocular disease**	**Iridoschisis (unilateral or bilateral)**	**Localization of iridoschisis**	**Other conditions**	**Complications after treatment**	**Associated cataract: Unilateral or bilateral**	**Phakic or pseudophakic**	**Glaucoma with symmetry or asymmetry of the optic nerve compared with the iridoschisis condition**	**Mechanical dilation or eye drops**
Smith et al. (2000)^(25)^	Journal of Cataract & Refractive Surgery	Case report	Female	88 years	1 year	No glaucoma	None reported	Bilateral	“The shredded-wheat appearance extended from 4 to 8 o’clock in both eyes.”	There were marked nuclear sclerotic changes in both lenses, as well as early retinal pigment epithelial changes at both maculae, compatible with the patient’s age.	No complications were mentioned.	Bilateral	Phakic	N/A	Tropicamide 1%, phenylephrine 10%, and diclofenac 0.1% drops are given four times in the hour before surgery to dilate the pupil and stabilize the BAB.
Wilczynski et al. (2013)^(26)^	European Journal of Ophthalmology	Case report	Female	66 years	N/A	Narrow-angle (she had a YAG iridotomy performed in both eyes a few years earlier)	She had a YAG iridotomy performed in both eyes owing to narrow-angle glaucoma a few years earlier.	Bilateral	Not specified, but in the right eye, areas of thinning of the iris stroma were visible	Nuclear and cortical opacities, anterior and posterior synechiae in the right eye	No complications	Bilateral	Phakic	N/A	Before the surgery, the pupil was dilated using a solution of 1% tropicamide and 10% phenylephrine (Neosynephrine); however, because of the posterior synechiae, the pupil did not dilate.
You et al. (2017)^(9)^	Medicine (Baltimore)	Case report	Female	67 years	3 months	Secondary glaucoma	None reported	Bilateral	Lower portion of the iris in both eyes	Secondary glaucoma (left eye), senile cataract (left eye), and pterygium (both eyes)	No complications	Unilateral (senile cataract in the left eye)	Phakic	N/A	Pupil dilation period was longer than the average pupil dilation time for patients with cataracts. Before LPI was conducted, four IOP-lowering drugs were prescribed (pilocarpine, timolol, brinzolamide, and brimonidine).

NA: not applicable.

**Table 2:** Clinical findings of the included studies

Study (Year)	Best-corrected visual acuity (BCVA: before and after treatment)	Intraocular pressure (IOP: before and after treatment)	Optic disc (before and after treatment)	Gonioscopy (before and after treatment)	Axial length	Endothelial cell count – pre e post op
Aaberg et al. (2017)^(14)^	N/A	Preoperative: 19 mm Hg (right eye) and 18 mm Hg (left eye)/Postoperative: N/A	Before: normal optic disc After: N/A	N/A	N/A	N/A
Auffarth (2005)^(13)^	Case 1: A preoperative BCVA of 0.05 in the right eye and 0.08 in the left eye. Postoperative not mentioned Case 2: A preoperative BCVA of 0.1 in the right eye and 0.3 in the left eye.	Case 1: 10 mm Hg in both eyes before and after treatment Case 2: N/A	Case 1: N/A. Case 2: N/A	N/A in both cases	N/A in both cases	N/A in both cases
Chen et al. (2017)^(4)^	Case 1: A clinical examination showed that the preoperative uncorrected visual acuities (UCVA) were hand movement OD and 20/40 for the OS. Furthermore, 2 months after surgery, the UCVA was 20/30 in the right eye and 20/25 in the left eye Case 2: UCVA before treatment was 20/200 OD and 20/200 OS. UCVA after treatment was 20/80 in the right eye 1 day postoperatively, improving to 20/50 1 month postoperatively.	Case 1: Preoperative: 13 mmHg in both eyes Postoperative (right eye): 1 day after surgery: 13 mmHg 1 week after surgery: 15 mmHg 1 month after surgery: 17 mmHg 2 months after surgery: 17 mmHg Postoperative (left eye): 1 day after surgery: 16 mmHg 1 week after surgery: 17 mmHg 1 month after surgery: 16 mmHg 2 months after surgery: 14 mmHg Case 2: Preoperative IOP was 12 mmHg in the right eye and 10 mmHg in the left eye. Postoperative IOP was 13mmHg in the right eye and not mentioned for the left eye.	Case 1: The right eye showed a mature cataract that hindered the visualization of the fundus and the evaluation of any optic disc alterations Case 2: The lens showed a mature cataract, which hindered the visualization of the fundus and the evaluation of any optic disc alterations.	N/A	N/A	Case 1: OD: Preoperative: 3.453 cells/mm2 Postoperative: 1 month after surgery: 1.085 cells/mm2 2 months after surgery: Not provided OS: Preoperative: 3.738 cells/mm^2^ Postoperative: 1 month after surgery: 2.630 cells/mm^2^ 2 months after surgery: 3.618 cells/mm^2^ Case 2: Preoperative endothelial cell count was 3.068 cells/mm^2^. Postoperative count was 1.456 cells/mm^2^.
Chen et al. (2023)^(15)^	Before: His BCVA was 0.4 (logMAR) in the right eye and 0.6 (logMAR) in the left After: 6 months after the surgery, the BCVA of both eyes increased by 0.1 (logMAR).	Before: At the time of admission, the IOP was 11.6 (OD)/11 (OS) mm Hg After: N/A	Before: The optic disc and macula appeared to be healthy in the posterior segment examination After: N/A	Before: Gonioscopy showed Shaffer Grade 4 angle (270°) in both eyes After: N/A	N/A	N/A
de Paula et al. (2011)^(5)^	Before: OD - 0.50, OS - 0.001 After: OD - N/A, OS - 1.0 (30 days postoperatively)	Before: OD - 14 mmHg, OS - 15 mmHg After: 14 mmHg in both eyes	Posterior segment examination indicated healthy optic discs and macula; changes not specified.	Before: Shaffer Grade 4 angle (270°) in both eyes After: N/A	OD: N/A, OS: 21.06 mm	N/A
Ghanem et al. (2003)^(16)^	BCVA before treatment was counting fingers in the OD and 20/100 in the OS. Postoperative BCVA improved to 20/25.	Preoperative IOP was 17 mmHg in the OD and 16 mmHg in the OS. Postoperative IOP was not mentioned.	N/A	Before: Gonioscopy was infeasible in the right eye. The left eye showed a slit angle without peripheral anterior synechia After: N/A	N/A	Preoperative endothelial cell count in the left eye was 1210 cells/mm^2^. Postoperative count was not specified.
Katipoğlu et al. (2023)^(17)^	OD: Preoperative: 0.1 (with correction), Postoperative (first day): 0.6, First-week and first-month follow-ups: 0.8 (+0.50–2.25 and 170) OS: Preoperative: 0.3, Postoperative (first day): Not provided, First-week and first-month follow-ups: Not provided	OD Preoperative: 13 mmHg, Postoperative (first day): 19 mmHg, First-week and first-month follow-ups: within normal limits OS: Preoperative: 14 mmHg, Postoperative (first day): Not provided, First-week and first-month follow-ups: within normal limits	Before: normal After: N/A	N/A	N/A	N/A
Lee et al. (2008)^(18)^	OD: Preoperative: 20/400, Postoperative (1 week after surgery): 20/100, 2 weeks postoperative day: 20/30, 1 month after surgery: 20/20 OS: Preoperative: 20/30, Postoperative: Not provided	OD: Preoperative: 15 mmHg, Postoperative (1 week after surgery): N/A, 2 weeks postoperative day: N/A OS: N/A	N/A	N/A	N/A	Preoperative: 2865 cells/mm^2^ in OD, not provided for the OS
Niu et al. (2024)^(19)^	Before treatment: OD: 20/80 OS: 20/30 After treatment: OD: 20/25 OS: 20/40	Preoperative: OD: 14 mmHg OS: 13 mmHg Postoperative: OD: 14 mmHg OS: 11 mmHg	N/A	N/A	OD: 25.39 mm OS: 25.45 mm	OD: Preoperative: 2946/mm^2^ Postoperative: 2278/mm^2^ OS: Preoperative: 2855/mm^2^ Postoperative: 2393/mm^2^
Omoto et al. (2021)^(20)^	Before treatment: OD: 20/600 OS: 20/30 After treatment: OD (post-DSAEK): 20/25 OS (post-DSAEK): 20/25	Preoperative: 10 mm Hg in both eyes Postoperative: Not provided	N/A	N/A	N/A	OD: Preoperative: Immeasurable. Postoperative: N/A OS: Preoperative: 1.197 cells/mm^2^. Postoperative: N/A
**Study (Year)**	**Best-corrected visual acuity (BCVA: before and after treatment)**	**Intraocular pressure (IOP: before and after treatment)**	**Optic disc (before and after treatment)**	**Gonioscopy (before and after treatment)**	**Axial length**	**Endothelial cell count – pre e post op**
Pieklarz et al. (2020)^(21)^	BCVA before treatment: “Hand motion” bilaterally BCVA after treatment: Improved to “counting fingers” in the OD and at least 20/200 in the OS.	IOP before treatment: 34 mmHg in the OD and 30 mmHg in the OS IOP after treatment: 2 weeks after the surgery, IOP was 17 mmHg (not specified)	Before: ‘’Severe optic disc damage found in our patient, particularly in the OS., suggested pre-existing glaucoma’’. The cup to disc (c/d) ratio was 0.9 with otherwise normal fundus in the left eye. In the OD, fundus examination showed c/d=0.6 and otherwise normal results After: N/A	N/A	22.76 mm for the OD and 22.68 mm for the OS.	Preoperative endothelial cell counts were 2412 cells/mm^2^ for the OD and 3189 cells/mm^2^ for the left eye Postoperative counts were not provided.
Porteous et al. (2014)^(22)^	Case 1: Before: BCVAs were 6/12 (OD) and 6/5 (OS), After: visual acuity of 6/6 in the right eye (OD), left eye not mentioned Case 2: Before: visual acuity of 6/36 (not specified), After: 6/12 (not specified)	Case 1: Before: IOP of 42 mmHg (OD) and 20 mmHg (OS). After: IOP of 18 mmHg (OD, the only one mentioned) Case 2: Before: IOP of 50 mmHg (OS, the only one mentioned), After: 14 mmHg (OS, the only one mentioned)	Case 1: Before: N/A. After: healthy optic discs Case 2: N/A	Case 1: Before: Indentation gonioscopy showed the iris strands to be appositional to the angle with no peripheral anterior synechiae. After: Healthy optic discs and open angles on gonioscopy Case 2: Before: N/A. After: the angles were open on gonioscopy.	Case 1: B-scan ultrasonography showed normal axial lengths (24.0/23.4 mm OD/OS) Case 2: N/A	N/A
Rozenberg et al. (2004)^(23)^	Before treatment: OD: 6/20, OS: 6/15 After treatment: OD: 6/7.5, OS: 6/6	Before treatment: 8 mm Hg in both eyes After treatment: N/A	N/A	N/A	N/A	N/A
Ruff et al. (2020)^(24)^	Before: Bare light perception in the OD and 20/150 in the OS After: OD: N/A, OS (the only submitted to surgery): 20/20 within 1 month of surgery	Before treatment: OD: 45 mm Hg. OS: 12 mm Hg After treatment: N/A	N/A	Before: On gonioscopic examination, the angle was obscured by iris strands in one quadrant, the rest of the angle seemed narrow After: N/A	OD: 22.41 mm. OS: 22.49 mm.	N/A
Smith et al. (2000)^(25)^	Before: ABCVA of 6/24 in both eyes After: Improved BCVAs to 6/9 postoperatively, and remained at 6/9 after 1 year	Preoperative: IOP was normal Postoperative: N/A	N/A	N/A	N/A	N/A
Wilczynski et al. (2013)^(26)^	Initial preoperative examination revealed a BCVA of 0.4 with correction −5.0 D in the OD and 1.0 with correction +4.5 D in the OS eye (the anisometropia amounted to 9.5 D) Postoperative: Postoperative uncorrected distance visual acuity after 1 week was 0.9. in the postoperative examination	Preoperative: In both eyes, the IOPs were within the normal range Postoperative: N/A	N/A	N/A	N/A	N/A
You et al. (2017)^(9)^	Before treatment: OD: 20/100 OS: Light perception After treatment: OD (2 days postoperation): 20/40 OD (1 month postoperation): 20/33 OS (1 month postoperation): Light perception OS (3 months postoperation): 20/33 OS (3 months postoperation): Light perception	Preoperative: OD: 22 mmHg OS: 58 mmHg Postoperative: OD: 16 mmHg OS: 44 mmHg	N/A	Before: Peripheral anterior synechiae were detected in three out of four quadrants via dynamic gonioscopy After: N/A	N/A	OD (preoperative): 2530 corneal endothelial cells/mm^2^ OS (preoperative): 1910 corneal endothelial cells/mm^2^ Postoperative of both eyes: N/A

Of the 21 patients analyzed, 14 (66.67%) exhibited iridoschisis in both eyes. Among those with a unilateral presentation (7 out of 21), the right eye was more affected, accounting for four of seven cases (57.71%). Among the studies included in this systematic review, the mean preoperative IOP in 28 eyes diagnosed with iridoschisis was 19.59±13.62mmHg (range: 8-58mmHg). Postoperatively, 15 eyes had a mean IOP of 16.46±8.09mmHg (range: 10-44mmHg). Progressive vision loss and blurring, followed by low BCVA, were the most common clinical features reported and were observed in 16 of the 21 cases. Other less common symptoms, such as eye pain, headaches, and changes in iris color, were reported in three cases.

The mean BCVAs for 16 left and 16 right eyes, as reported in the studies, were as follows: (OD: 0.21±0.20; OS: 0.27±0.32; Mean±SD). Postoperatively, for the studies that explicitly reported this clinical characteristic, the mean BCVAs for 10 right eyes and 11 left eyes were as follows: (OD: 0.69±0.28; OS: 0.64±0.34; Mean±SD). All parameters were standardized to a decimal scale using the established transformation methods in the literature.^( 1 , 2 , 3 )^ Seven articles reported endothelial cell count (ECC). The preoperative ECC measurements in 11 eyes with iridoschisis averaged 2,573±838.65 cells/mm^2^ (range: 1910-3,738 cells/mm^2^). The postoperative mean ECC from four eyes with iridoschisis was 2,096±690.07 cells/mm^2^ (range: 1,085-2,630 cells/mm^2^).

Six studies reported the condition of the optic disc before treatment, resulting in the analysis of 15 eyes. Chen et al. demonstrated that the presence of mature cataracts impeded the evaluation of the optic disc in both cases reported. Moreover, Pieklarz et al. observed severe optic disc damage in both eyes with iridoschisis. The other four studies conducted by Aaberg et al., Chen et al., Ghanem et al., and Katipoğlu et al. reported healthy optic disc visualization in the examined eyes.

The presence of secondary glaucoma owing to iridoschisis was directly reported in four studies, involving five of seven (71.42%) patients diagnosed with this condition. The remaining two cases were linked to chronic glaucoma. Upon analyzing the ocular medical history documented in each study, 20 (95.23%) of the 21 patients exhibited some cataract manifestations in the same eye affected by iridoschisis. In only one case reported by Chen et al., the patient had a mature cataract in the eye contralateral to that with iridoschisis. Nineteen of the 21 patients retained their natural crystalline lenses (phakic); the other two had previously undergone intraocular lens implantation. Notably, the participants described in the studies by Chen et al. and Lee et al. underwent cataract surgery following iridoschisis diagnosis.

Cataract surgery was the most common treatment and was performed using diverse surgical techniques, depending on the patient’s condition, in all analyzed cases. Only lens extraction and intraocular lens (IOL) implantation were performed in the patient’s right eye, as reported by Pieklarz et al., and no cataract surgery was performed. Corneal edema was the most frequent post-treatment complication reported in the studies, occurring in three patients. Other common complications included a moderate inflammatory response, Descemet folds, and fibrillary material deposition on the lens surface, each present in two cases. Finally, less common complications such as high postoperative cylindrical refractive error, the suspicion of uveitis glaucoma hyphema syndrome, and equivocal iris changes were reported in one case each.

### Evaluating the risk of bias

Table 1 presents the risk of bias evaluated using the Critical Appraisal Checklist for Case Reports. The first assessment criterion was patient demographics. All studies included information about sex, age, and, in some cases, employment status (Yes: 15). The second factor was patient history and timelines; five of the 15 studies did not include these (Yes: 10, No: 5). The third criterion was an accurate description of the patients’ present clinical status, with two studies of 15 not considering these (Yes: 13, No: 2). The fourth criterion was a detailed explanation of the intervention and therapy process; all 15 studies addressed this in-depth (Yes: 15). The fifth assessment criterion was a clear explanation of the intervention and therapy processes; in all 15 investigations, broad remarks were given (Yes: 15). The sixth criterion requested a detailed account of post-intervention clinical circumstances; in all studies after ocular surgery, the clinical condition improved without significant complications, with a good response to treatment (Yes: 15). The seventh criterion was information on unforeseen or unfavorable occurrences; only two studies did not explain this (Yes: 13). The eighth criterion was the provision of takeaway lessons by the case reports; all studies were deemed beneficial because they covered multiple diseases associated with iridoschisis (cataract and glaucoma) in addition to the various surgical access methods and treatments used (phacoemulsification with iris hook, stromal puncture, and Malyugin ring).

Table 2 summarizes the bias risk evaluated using the Critical Appraisal Checklist for Case Series. The first assessment criterion was that there were clear criteria for inclusion in the case series, as it is a rare disease; this criterion is Not Available for our study (NA: 2). The second assessment criterion evaluated whether the condition was measured in a standard, reliable manner for all patients in all cases. Despite the rarity of iridoschisis, the articles reached a conclusion about this disease after excluding other differential diagnoses (Yes: 2). The third assessment criterion was valid methods used to identify the condition for all patients included in the case series; iridoschisis in the studies was considered after excluding the differential diagnosis (Yes: 2). Fourth, the case series included consecutive participants. The studies had small population sizes owing to the rarity of iridoschisis; therefore, this criterion was not applied in our study (NA: 2). The fifth requirement was that this case series had a complete inclusion of participants. For the same reason explained in the fourth question of the checklist, this criterion did not apply in our study (NA: 2). The sixth criterion was a detailed report of the demographics of the study participants; all studies included information about age and sex (Yes: 2). The seventh criterion was a clear report of the clinical information of the patients;, all studies reported the stage of the disease using ophthalmological examinations, comorbidities, and the stage of iridoschisis (Yes: 2). The eight criterion was the comprehensive report of outcomes or follow-up of cases; all studies included these (Yes: 2). The ninth requirement was clearly reporting the presenting site or clinical demographic information; all studies reported the prevalence and diseases associated with iridoschisis (Yes: 2). The tenth criterion was suitability for statistical analysis; this requirement was not met, and there was no statistical selection of cases (NA: 2).

## DISCUSSION

Iridoschisis is a rare bilateral condition associated with trauma or previous surgeries.^( 1 )^ It is estimated that there are more cases of iridoschisis than previously reported. This is possibly owing to the limited knowledge and challenges in diagnosing dark-colored irises.^( 18 )^ Therefore, the multimodal imaging evaluation of the affected patients is essential for the correct diagnosis and management (surgical or clinical) of glaucoma.

We present a unilateral case in which the patient had no prior ophthalmological intervention, and the diagnosis was confirmed following imaging. Preoperative planning of the facectomy was performed considering the possible intraoperative surgical challenges. In addition, the correct diagnosis facilitated the investigation of glaucomatous optic neuropathy. This is a common sight-threatening condition that requires early diagnosis and treatment, as it largely compromises the patient’s visual acuity.

This case emphasizes the relevance of correct diagnosis in managing glaucoma and allowing the planning of a facectomy by experienced surgeons owing to possible intraoperative difficulties.^( 5 )^ In addition, multimodal analysis enables complete patient assessment by integrating iridoschisis findings in all ocular segments.

In our systematic review, we analyzed 21 patients with iridoschisis associated with cataracts, involving 35 eyes. Iridoschisis was predominantly bilateral and affected women more frequently; the mean age was 66.95 years. The mean preoperative and postoperative IOP values were 19.59 and 16.46mmHg, respectively. The mean BCVA improved significantly after surgery. Preoperative ECC measurements were taken in 11 eyes with iridoschisis, averaging 2573±838.65 cells/mm^2^, while postoperative ECC values in four eyes averaged 2096±690.07 cells/mm^2^.

Iridoschisis is associated with various ocular conditions, including angle-closure glaucoma and cataracts. Although Danias et al. suggested a possible hereditary nature of iridoschisis, sporadic cases are common.^( 27 )^ The management of glaucoma usually involves laser iridotomy; however, goniosynechialysis combined with cataract removal may be more effective in cases of angle closure induced by peripheral anterior synechiae.^( 28 )^ In our review, the management of glaucoma varied across studies, with a combination of medical and surgical interventions, often tailored to an individual patient’s condition. Cataract surgery, along with glaucoma management, showed favorable outcomes in terms of IOP control and visual acuity improvement.^( 29 , 30 )^

Our results confirm the effectiveness of phacoemulsification with IOL implantation in improving BCVA in patients with iridoschisis, as reported by Minezaki et al. and Greenwald et al.^( 28 , 31 )^ Non-Descemet stripping automated endothelial keratoplasty and Descemet membrane endothelial keratoplasty showed promising results in treating corneal decompensation secondary to iridoschisis. However, cataract surgery in patients with iridoschisis requires additional precautions owing to the risk of aspirating the iris fibrils and other intraoperative complications. Strategies such as the use of dispersive viscoelastics and pupil expanders, including the Malyugin ring, are recommended.^( 26 )^ In addition, the excision of floating iris fibers with microcauterization has been suggested as an effective technique.^( 6 )^ In our review, the adopted techniques had varying degrees of success, indicating that while they are effective, the optimal technique should be individualized based on patient-specific factors.^( 32 , 33 )^

The reduction in postoperative ECC in our review is consistent with that of other studies, indicating that surgery improves visual acuity and impacts endothelial health. This highlights the significance of careful preoperative and postoperative management to preserve endothelial function optimally.

The choice of the surgical technique significantly influences the outcomes of patients with iridoschisis. In our review, various techniques were used, and the outcomes suggest that while no single technique is superior in all cases, the selection should be based on the specific clinical scenario, the surgeon’s expertise, and available resources.

This study had some limitations. The primary limitation was that the included studies were case reports and case series, leading to high heterogeneity among the cases. Most data came from case reports, which complicates generalizing the results. Additionally, the small patient population and variability in clinical characteristics and treatments pose significant challenges. The lack of long-term follow-up in many studies hindered the assessment of the long-term stability of surgical outcomes. There is no standardized surgical technique, and the reported outcomes are variable.

## CONCLUSION

This case and systematic review provide valuable insights into managing iridoschisis that is associated with cataract and its comorbidities. The study emphasizes the importance of multimodal imaging in patients with iridoschisis for accurate diagnosis and identification of associated diseases. Multimodal imaging has proven effective in the early detection and management of this infrequently reported complication. Glaucoma is common in these patients and, if not treated, results in adverse outcomes. In addition, the knowledge and diagnosis of this condition are crucial in the surgical planning of facectomy, because of the possible intraoperative risks and difficulties. Finally, understanding differential diagnoses is vital, and iridocorneal endothelial and Axenfeld-Rieger syndromes should always be excluded. Although current treatment approaches are effective in improving visual acuity and managing intraocular pressure, the recurrence of glaucoma and other complications highlights the need for ongoing research to refine treatment strategies and improve the quality of care. Randomized and long-term studies are warranted to evaluate disease- and surgical treatment-related factors and their impacts on anatomical and visual outcomes.

DATA AVAILABILITY:

The underlying content is contained within the manuscript.
